# Exploratory Multimodal Analysis of Vascular Changes in Basal Cell Carcinoma Before and After Topical Imiquimod Therapy Using Dermoscopy and Non-Invasive Imaging 

**DOI:** 10.3390/cancers18132153

**Published:** 2026-07-04

**Authors:** Oliver Mayer, Hanna Wirsching, Sophia Schlingmann, Deborah Winkler, Lena Schemet, Tobias Kaps, Julia Welzel, Sandra Schuh

**Affiliations:** 1 Department of Dermatology and Allergology, University Hospital Augsburg, 86156 Augsburg, Germany; hanna.wirsching@uk-augsburg.de (H.W.); sophia.schlingmann@uk-augsburg.de (S.S.); deborah.winkler@uk-augsburg.de (D.W.); julia.welzel@uk-augsburg.de (J.W.); sandra.schuh@uk-augsburg.de (S.S.); 2 Chair of Mathematical Statistics and Artificial Intelligence in Medicine, University of Augsburg, 86159 Augsburg, Germany; lena.schemet@uni-a.de (L.S.); kaps.tobi@web.de (T.K.)

**Keywords:** basal cell carcinoma, imiquimod, vascular changes, dermoscopy, dynamic optical coherence tomography (D-OCT), line-field confocal optical coherence tomography (LC-OCT), microcirculation, exploratory imaging, non-invasive imaging

## Abstract

**Background/Objectives:** Topical imiquimod is an established non-invasive treatment for superficial basal cell carcinoma (sBCC). However, data on treatment-associated changes in tumor microvascularization remain limited. This study investigated vascular changes before and after imiquimod therapy using multimodal non-invasive imaging. **Methods:** In this single-center, prospective observational study, 31 basal cell carcinomas in 20 patients were examined before and 12–16 weeks after topical imiquimod therapy (5%, five times weekly for six weeks) using dermoscopy, dynamic optical coherence tomography (D-OCT), and line-field confocal optical coherence tomography (LC-OCT). Analyses were performed as paired before-and-after comparisons. While approved for sBCC, a small number of thin nodular and infiltrative BCCs were included exploratorily; subgroup analyses were not powered. **Results:** Dermoscopy showed a nominally significant shift toward smaller vessel diameter categories after therapy (ATS = 8.183, df = 1, *p* = 0.004). D-OCT-derived parameters (vessel density, vessel diameter, and depth of the vascular plexus) did not show nominally significant changes. LC-OCT showed nominally lower apparent intratumoral flow scores (ATS = 13.285, df = 1, *p* < 0.001), reduced occurrence of vessel-wall-associated intraluminal structures showing a rolling-like motion pattern (86.7% before treatment versus 33.3% after treatment; ATS = 13.357; df = 1, *p* < 0.001), and a reduction in maximum vessel diameter (ATS = 6.110, df = 1, *p* = 0.013). The primary LC-OCT inferential analyses were performed at the lesion level without adjustment for within-patient clustering and should therefore be interpreted as exploratory. An additional patient-cluster-adjusted paired change-score sensitivity analysis for LC-OCT maximum vessel diameter yielded a directionally consistent estimate (−17.81 µm; 95% CI: −34.40 to −1.23; *p* = 0.037). The primary exploratory endpoints were LC-OCT–based apparent intratumoral flow and maximum vessel diameter; secondary endpoints included dermoscopic and D-OCT–based vascular parameters. In the exploratory response-stratified analysis, the change in LC-OCT-based maximum vessel diameter did not differ significantly among the assigned response groups (Kruskal–Wallis H = 3.870, df = 2, raw *p* = 0.144; BH-adjusted *p* = 0.753). **Conclusions:** LC-OCT detected several exploratory vascular changes between the pre-treatment examination and follow-up and may provide complementary information for the non-invasive assessment of BCC after imiquimod therapy. Given the exploratory design, limited sample size, and lack of systematic histological confirmation, these findings are hypothesis-generating and require validation in larger prospective studies.

## Simple Summary

Basal cell carcinoma is the most common type of skin cancer. In selected superficial cases, it can be treated without surgery using topical imiquimod cream. However, it is still unclear how the small blood vessels within and around these tumors change during treatment. In this prospective study, we examined basal cell carcinomas before and after imiquimod therapy using three non-invasive imaging techniques: dermoscopy, dynamic optical coherence tomography, and line-field confocal optical coherence tomography. Line-field confocal optical coherence tomography detected several exploratory changes in apparent intravascular motion and vessel morphology between the pre-treatment examination and follow-up. These findings may contribute to a better understanding of vascular changes after topical treatment but require validation before clinical use in treatment monitoring can be considered.

## 1. Introduction

Basal cell carcinoma (BCC) is the most common malignant skin tumor in fair-skinned populations [1,2]. For superficial BCC, in addition to surgical excision, established non-invasive treatment options such as topical imiquimod are available [1]. While the morphological effects of treatment are well documented, data on tumor-associated microvascular changes before and after topical imiquimod therapy remain limited [3]. Recent work on artificial intelligence-assisted dermatologic screening has additionally highlighted the epidemiological burden and heterogeneous clinical presentation of BCC and illustrates the increasing role of advanced technologies in non-invasive skin cancer assessment [4].

Superficial BCC (sBCC) generally has a low-risk profile, particularly in small primary lesions at non-critical sites such as the trunk or extremities [1]. In addition to standard surgical excision, the German S2k guideline also recommends non-invasive treatment options such as photodynamic therapy, topical therapy with 5-fluorouracil (5-FU), or imiquimod 5% [1,5,6]. In Europe, imiquimod 5% is approved for use in immunocompetent adults for sBCC with a lateral extent of <2 cm [1]. Its efficacy has been demonstrated in randomized, vehicle-controlled Phase III studies [7,8].

By activating Toll-like receptor 7 (TLR7) as a selective agonist, imiquimod triggers a local pro-inflammatory immune response in the skin. Banzhaf et al. demonstrated structural changes including reduced tumor thickness, morphological alterations, and regression of BCC-specific structures before and after completion of imiquimod therapy using OCT [9].

In 2021, Verzì et al. visualized residual tumor structures following imiquimod treatment due to the high spatial resolution of LC-OCT. However, systematic quantitative and qualitative analyses of vascular changes were not performed in these studies [3].

Dermoscopy is a key tool for assessing vascular patterns [10]. Dynamic optical coherence tomography (D-OCT) enables the in vivo visualization of blood flow structures using speckle variance analysis [11]. The VivoSight Dx from Michelson Diagnostics (Maidstone, Kent, UK) allows for the determination of parameters such as vascular density, vessel caliber, and the depth of the subepidermal vascular plexus [11,12]. The deepLive™ line-field confocal optical coherence tomography (LC-OCT) from DAMAE Medical (Paris, France) is an advancement in high-resolution non-invasive skin imaging [13]. Donelli et al. have already demonstrated its high diagnostic accuracy for BCC [14].

Although OCT and LC-OCT can visualize treatment-associated changes in BCC, systematic multimodal analyses of vascular changes during standardized imiquimod therapy remain scarce [3,9]. A combined approach using dermoscopy, D-OCT, and LC-OCT may therefore provide complementary information.

The aim of this study was to investigate vascular changes in basal cell carcinomas before and after topical imiquimod therapy using in vivo multimodal non-invasive imaging with dermoscopy, D-OCT, and LC-OCT, and to explore whether selected vascular imaging parameters differed across the assigned clinical/imaging response categories.

## 2. Materials and Methods

### 2.1. Study Design and Ethics

In this single-center, prospective observational clinical study, lesions were examined using dermoscopy, D-OCT, and LC-OCT before treatment and 12–16 weeks after completion of imiquimod therapy.

Follow-up was scheduled after the acute inflammatory reaction had subsided in order to assess therapy-associated vascular changes independently of short-term inflammatory effects. The exact timing varied within this interval for practical reasons.

Routine post-treatment histological examination was not included because the study protocol was designed as a fully non-invasive follow-up approach. In lesions that were clinically and non-invasively unremarkable, biopsy was not routinely performed in the absence of a predefined clinical indication, as this would have introduced additional invasiveness. However, this design precluded histological confirmation of treatment response and did not allow subclinical residual tumor to be excluded. Accordingly, the imaging findings cannot be interpreted as evidence of histologically confirmed tumor clearance or used to determine diagnostic accuracy for residual disease. The broader feasibility of reducing invasive procedures in selected BCC diagnostic pathways has been demonstrated in a randomized trial of OCT-guided diagnosis and treatment [15]. However, that study does not eliminate the need for an independent reference standard when histologically confirmed post-treatment clearance is the outcome of interest.

The study was conducted at the Department of Dermatology and Allergology of the University Hospital Augsburg following approval by the Ethics Committee of LMU Munich (project numbers: 17-0699 and 22-0781).

The primary objective of the study was to assess pre- and post-treatment changes in LC-OCT-based vascular parameters of basal cell carcinomas under topical imiquimod therapy, with a focus on apparent intratumoral flow and maximum vessel diameter as exploratory primary endpoints. Secondary endpoints included dermoscopic vascular features, D-OCT-derived vascular parameters including vessel density, vessel diameter, and plexus depth, the occurrence of vessel-wall-associated intraluminal structures showing a rolling-like motion pattern, and additional LC-OCT-derived structural parameters.

### 2.2. Study Population

A total of 20 patients (9 females, 11 males; mean age 72.3 years; median 73 years; SD 8.6 years; range 51–93 years) with BCC confirmed either histologically or by non-invasive diagnostic methods were included in the study. In lesions without pre-treatment histology, both diagnosis and subtype assessment were based on established non-invasive imaging criteria using D-OCT and, where required, LC-OCT; therefore, subtype classification should be interpreted as histological where available and imaging-based otherwise.

Apart from a diagnostic biopsy, performed in five tumors, prior treatment of the target BCC before study inclusion was not permitted.

With regard to tumor location, there were no general exclusion criteria provided that imaging of sufficient quality and topical treatment with imiquimod were considered clinically feasible; lesions in the immediate vicinity of the eye were excluded. Treatment of nodular and infiltrative BCCs was performed as an exploratory off-label approach.

Superficial, nodular, and infiltrative BCCs were eligible if the maximum tumor thickness was ≤2 mm.

Tumor thickness was determined histologically, where available; for purely imaging-based (OCT-based) assessments, only lesions with a maximum tumor thickness of 1.2 mm were included.

In the absence of pre-treatment histology, assessment was based on D-OCT and supplemented by LC-OCT when tumor margins were unclear. Only lesions meeting the predefined thickness criteria based on combined imaging and clinical evaluation were included.

For an exploratory analysis, the clinical response to therapy was coded as 0 = no clinical/imaging response, 1 = partial clinical/imaging response, and 2 = complete clinical/imaging response. This exploratory response classification was not based on an independent blinded reference standard and partly incorporated imaging findings. Because imaging findings contributed both to the assigned response category and to the imaging-derived study variables under investigation, the response-stratified analysis is susceptible to incorporation bias. The classification was therefore used only for descriptive, hypothesis-generating comparisons and not for evaluating diagnostic or predictive performance.

### 2.3. Treatment with Imiquimod 5%

Imiquimod 5% cream was applied five times weekly for six weeks, using the dosing regimen approved for superficial BCC. In nodular and infiltrative BCCs, the same regimen was used on an exploratory off-label basis [1,7].

Patients received standardized instructions regarding treatment application and the management of local inflammatory reactions. Application was not formally monitored or quantified. Erythema and crust formation were documented at follow-up but represented only indirect, non-standardized indicators of adherence. Skincare products were permitted but were not applied simultaneously with imiquimod.

### 2.4. Dermoscopy

Dermoscopy was performed using the videodermoscope attached to the LC-OCT device (deepLive™) from DAMAE Medical (Paris, France), utilizing both polarized and non-polarized light. Regardless of the type of illumination, an immersion oil (paraffin oil) was used to reduce surface reflections.

The presence of punctate, globular, linear, serpiginous, or curved vessels was assessed. Furthermore, blood vessel orientation, density, and distribution were analyzed [10,16,17,18].

The dermoscopic vessel diameter was recorded semiquantitatively on an ordinal scale.

### 2.5. D-OCT

The VivoSight Dx from Michelson Diagnostics (Maidstone, Kent, UK) was used in the study. This device, equipped with a 1305 nm laser source, has an axial resolution of <5 µm and a lateral resolution of <7.5 µm [19,20].

Vascular patterns can be visualized using speckle variance analysis based on the movement of individual blood cells [12].

Furthermore, the device allows for the automatic calculation of vascular density, vessel diameter, and the depth of the subepidermal vascular plexus [11,19,21].

In our study, the images were standardized and evaluated at a depth of 300 µm and were only included if the quality allowed for a sufficient evaluation. Images with hyperkeratosis-related shadowing, motion, or pressure artifacts were excluded.

### 2.6. LC-OCT

The LC-OCT deepLive™ has a lateral resolution of <1.3 µm at a maximum penetration depth of 500 µm. It combines confocal laser microscopy with conventional OCT [13].

LC-OCT was primarily used as a high-resolution morphological imaging modality. In addition, horizontal video sequences were explored for the semiquantitative assessment of dynamic intravascular features.

The sequences were evaluated using a horizontal mode, during which the diameter of the largest clearly distinguishable vessel; the diameter of intraluminal cells in the vessel center and at the vessel wall was determined. Apparent intratumoral flow was assessed semiquantitatively on a four-point ordinal scale (0 = no visible movement; 1 = low flow; 2 = moderate flow; 3 = high flow), based on the visual impression of intraluminal motion [22,23,24]. These parameters were interpreted as exploratory video-based indicators of intravascular motion rather than validated quantitative perfusion measurements. For the purpose of this exploratory analysis, vessel-wall-associated intraluminal structures showing a rolling-like motion pattern were recorded as absent or present. This imaging phenotype was defined as clearly distinguishable, slowly moving cell-like structures observed in close contact with the vessel wall over consecutive frames. The classification was based on wall-adjacent location, apparent size and movement characteristics. LC-OCT morphology alone does not permit definitive cellular identification. Therefore, these structures should not be interpreted as histologically or immunohistochemically confirmed leukocytes. Alternative intraluminal structures, cellular debris, aggregates, flow-related phenomena, or imaging artifacts cannot be excluded. The presence of inflammatory cells in the interstitium was also documented.

Horizontal LC-OCT video sequences lasted approximately 10–25 s and were acquired with minimal probe pressure.

For analysis, representative segments of each sequence were selected based on image stability, focus, and absence of motion artifacts. If multiple sequences were available, the sequence with the highest image quality was used.

### 2.7. Image Acquisition & Analysis

If the lesions lacked suitable landmarks, the measurement area was marked on the skin, followed by photographic documentation.

Image acquisition was performed by trained investigators. To avoid pressure-dependent perfusion changes, all devices are designed for minimal compression [11,13,21].

One trained investigator (O.M.), who was aware of the examination time point, performed the primary image evaluation. Selected datasets were reviewed by a second experienced investigator (S.S.) for plausibility and consistency only; this review was neither systematic nor blinded and did not constitute independent second-reader validation. Response classification and imaging-parameter assessment were also not fully independent, creating potential observer and incorporation bias. No formal interobserver, intraobserver, or test–retest analysis was performed. Due to modality-specific quality requirements, not all lesions were evaluable for every parameter. The number of included datasets therefore varied between analyses and is reported for each parameter.

### 2.8. Statistics

Statistical analyses were exploratory, and no adjustment for multiple testing was performed for the main pre–post analyses. Accordingly, *p*-values < 0.05 were interpreted as nominally significant. No imputation of missing data was performed. Analyses were based on available cases, and the number of evaluable lesions varied by endpoint.

Descriptive statistics were used to summarize patient-, lesion-, and imaging-related characteristics. Categorical and ordinal variables are presented as absolute and relative frequencies, whereas metric variables are summarized descriptively using appropriate measures of central tendency and dispersion.

Paired lesion-level pre-treatment and follow-up comparisons were performed using rank-based methods implemented with the R package nparLD and the function nparLD::ld.f1(). This corresponds to an LD-F1 design with one within-unit factor, timepoint, comprising two levels (pre-treatment and follow-up), and no additional between-unit grouping factor. The lesion was used as the paired observational unit. For the principal inferential results, the ATS, corresponding degrees of freedom, and two-sided *p*-value were reported. Because two timepoints were compared, the degrees of freedom for the Time effect were df = 1. These analyses account for the pre–post pairing within lesions but do not additionally account for clustering of multiple lesions within the same patient.
For four continuous imaging endpoints with sufficient paired data, additional patient-cluster-adjusted change-score sensitivity analyses were performed. For each lesion, the change score was calculated as Δ = follow-up minus pre-treatment value. The models were fitted using lmerTest::lmer(delta ~ 1 + (1 | patient_id), REML = TRUE). Ninety-five percent confidence intervals were calculated as Estimate ± $t_{0.975,df}$ × SE, using the endpoint-specific denominator degrees of freedom provided by lmerTest. *p*-values were obtained from summary(fit)$coefficients and therefore correspond to the lmerTest/Satterthwaite output. Model singularity was assessed using lme4::isSingular(), with the tolerance set to 1 × 10^−5^. Singular or boundary fits were reported transparently and interpreted cautiously.
These patient-cluster-adjusted change-score models were performed as sensitivity analyses and did not replace the main lesion-level paired rank-based pre–post analyses. The *p*-values reported in the main endpoint tables therefore refer to the lesion-level LD-F1 analyses performed using nparLD::ld.f1(). The mixed-effects model estimates, 95% confidence intervals, *p*-values, and model-status information are reported separately in Supplementary Table S1. The D-OCT plexus-depth model showed a boundary/singular fit, with the patient-level random-intercept variance estimated near zero, whereas the models for D-OCT vessel density, D-OCT vessel diameter, and LC-OCT maximum vessel diameter converged without a singular fit. Results from singular or boundary models were interpreted cautiously.

LC-OCT apparent intratumoral flow was treated as an ordinal/categorical endpoint rather than as a binary 0/1 outcome. Therefore, a binary logistic mixed-effects model was not appropriate for this endpoint coding. Although an ordinal mixed-effects model would in principle have been the corresponding model class, such a model was not pursued as a robust sensitivity analysis because instability was expected given the small sample size and sparse or unevenly distributed ordinal categories. This endpoint was therefore reported using the lesion-level rank-based/ordinal analysis.

Apart from the four continuous endpoints listed above, the dermoscopic endpoints and the remaining semiquantitative, qualitative, and structural imaging endpoints for which no cluster-adjusted sensitivity model was fitted were analyzed at the lesion level without additional adjustment for patient-level clustering. These unadjusted analyses may underestimate uncertainty and yield anti-conservative *p*-values and are therefore interpreted as exploratory. The statistical method was selected according to the measurement scale of the respective endpoint, the structure of the available data, and model quality. The results of all four continuous change-score models are reported in Supplementary Table S1.

For the binary endpoint describing vessel-wall-associated intraluminal structures showing a rolling-like motion pattern, a generalized linear mixed-effects model with a logit link, time point as a fixed effect, and a random intercept for patient was initially explored. Because this model resulted in a singular/boundary fit in the presence of sparse and unbalanced data, the mixed-model odds ratio and confidence interval were not used for inferential interpretation. The endpoint was instead reported using the observed pre- and post-treatment frequencies and the lesion-level rank-based paired analysis. No regression-derived odds ratio or confidence interval is reported for this endpoint.

Exploratory response-group analyses were performed at the lesion level. For each endpoint, global comparisons among the three assigned response groups—no clinical/imaging response, partial clinical/imaging response, and complete clinical/imaging response—were performed separately for pre-treatment values, follow-up values, and lesion-level change scores using the Kruskal–Wallis test (stats::kruskal.test). Change was defined as Δ = follow-up minus pre-treatment value. For each of the three analysis sets (pre-treatment, follow-up, and Δ), the global *p*-values were adjusted across the evaluated endpoints using the Benjamini–Hochberg method. Exploratory pairwise comparisons of the Δ values were performed using pairwise.wilcox.test(..., p.adjust.method = “BH”, exact = FALSE), with adjustment across the three pairwise group comparisons within each endpoint. Raw and BH-adjusted *p*-values are reported. These response-group analyses were not additionally adjusted for clustering of multiple lesions within patients and may therefore underestimate uncertainty. Given the small and markedly unbalanced response groups, all response-stratified analyses were interpreted descriptively and as hypothesis-generating.

Statistical analyses were performed using R version 4.2.2.

## 3. Results

### 3.1. Patient and Lesion Characteristics

The analysis included 20 patients with a total of 31 basal cell carcinomas. Several patients had more than one lesion. The majority of the tumors were sBCC (76.7%), followed by nodular (20.0%) and one infiltrative BCC (3.3%) (Table 1). Although pre-treatment and follow-up examinations were performed for all included lesions, the number of evaluable paired measurements varied by modality and endpoint because of image-quality exclusions and missing endpoint-specific information; the corresponding paired n is reported for each analysis.

Additional lesion-level baseline characteristics, including lesion size, anatomical location, and clinical risk zone classification, are summarized in Table 1. Information on pigmentation and ulceration was recorded where available.

**Figure 1:**
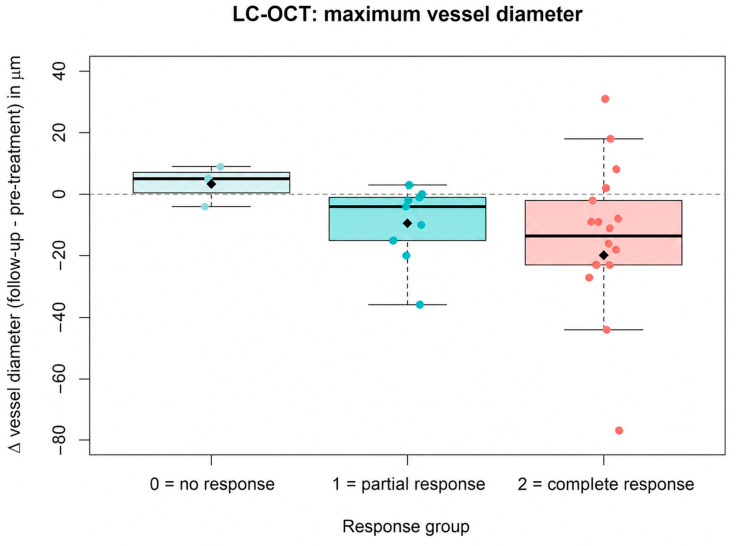
LC-OCT-based change in maximum vessel diameter as a function of clinical response to therapy. The figure shows the change in maximum vessel diameter (Δ = post-treatment follow-up − pre-treatment; µm) for lesions with no clinical/imaging response (0), partial clinical/imaging response (1), and complete clinical/imaging response (2). Each point represents a lesion; the box plots show the median and interquartile range. Negative Δ values correspond to a decrease in vessel diameter at follow-up. The global Kruskal–Wallis comparison of the Δ values was not statistically significant (H = 3.870, df = 2, raw *p* = 0.144; BH-adjusted *p* = 0.753), and all exploratory pairwise Δ comparisons were also non-significant (BH-adjusted *p* ≥ 0.173). The displayed group pattern is descriptive only and should not be interpreted as evidence that vessel-diameter reduction tracks or predicts treatment response. Response classification partly incorporated imaging findings and was not based on an independent blinded reference standard. Each point represents one lesion (*n* = 30); only lesions with available paired LC-OCT measurements were included.

In the exploratory response-oriented analysis, response categories were available for 30 lesions: no clinical/imaging response, *n* = 3; partial clinical/imaging response, *n* = 9; complete clinical/imaging response, *n* = 18. Selected lesion-level changes in LC-OCT maximum vessel diameter and rolling-like status across the assigned response groups are summarized in Table 2. No statistically significant between-group differences remained after Benjamini–Hochberg adjustment for any of the evaluated pre-treatment, follow-up, or Δ values. For LC-OCT maximum vessel diameter, the global comparison of Δ values yielded a Kruskal–Wallis statistic of H = 3.870 with df = 2, a raw *p*-value of 0.144, and a BH-adjusted *p*-value of 0.753. Exploratory pairwise Δ comparisons were also non-significant, with BH-adjusted *p*-values of 0.173 for no versus partial response, 0.173 for no versus complete response, and 0.381 for partial versus complete response. Descriptively, the median change was +5.0 µm [IQR 0.5 to 7.0] in lesions classified as showing no response, −4.0 µm [IQR −15.0 to −1.0] in lesions showing a partial response, and −13.5 µm [IQR −23.0 to −3.5] in lesions showing a complete response (Figure 1 and Supplementary Table S2). This numerical pattern is descriptive only and should not be interpreted as evidence that vessel-diameter reduction is associated with, predicts, or validly tracks treatment response.

### 3.2. Dermoscopic Before-After Analysis

**Figure 2:**
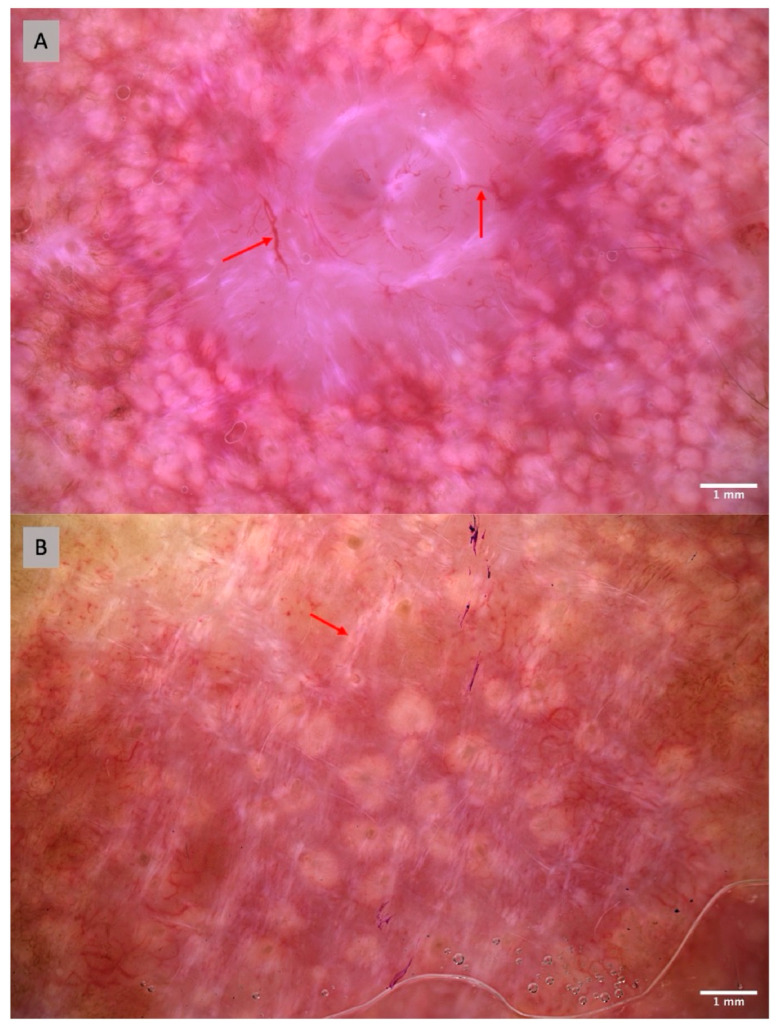
Dermoscopic images of treatment-associated vascular changes in a basal cell carcinoma before and after topical imiquimod therapy. (**A**) Pre-treatment dermoscopic image of a nodular basal cell carcinoma with prominent tumor-associated vascular structures, including linear and arborizing vessels (red arrows). (**B**) 16 weeks after completion of imiquimod therapy, the previously visible vessels of the same BCC appear narrowed and less prominent, consistent with regression of superficial vascular tumor structures.

In the before-after comparison, a nominally significant time effect was observed, with a shift in the ordinal distribution toward smaller-vessel diameter categories following imiquimod therapy (ATS = 8.183, df = 1, *p* = 0.004; Figure 2). The analysis accounted for the pre–post pairing within lesions but not for clustering of multiple lesions within patients; the *p*-value may therefore be anti-conservative and is interpreted as exploratory.

### 3.3. D-OCT-Based Vascular Parameters

Vascular density, vessel diameter, and the depth of the subepidermal vascular plexus were analyzed before and after treatment using D-OCT. The primary lesion-level paired rank-based analyses did not demonstrate nominally significant pre–post changes for any of these parameters. The metric D-OCT parameters are summarized in Table 3, the semiquantitative D-OCT parameters in Table 4, and the qualitative vascular morphology findings in Table 5.

In additional cluster-adjusted paired change-score sensitivity analyses, the estimated mean change in D-OCT plexus depth was 159.83 µm (95% CI: −115.40 to 435.07; *p* = 0.245; 30 paired lesions from 20 patients). This model had a boundary/singular fit, with the patient-level random-intercept variance estimated near zero, and should therefore be interpreted cautiously.

For D-OCT vessel density, the estimated mean change was 1.48 percentage points (95% CI: −3.19 to 6.16; *p* = 0.515; 30 paired lesions from 20 patients), and the model converged. For D-OCT vessel diameter, the paired change-score model yielded an estimated mean change of 7.94 µm (95% CI: −12.53 to 28.42; *p* = 0.428; 26 paired lesions from 19 patients). The model converged. No evidence of a statistically significant pre–post change was observed.

These sensitivity analyses did not provide evidence of statistically significant changes in the investigated D-OCT vascular parameters after accounting for patient-level clustering. The detailed results are reported in Supplementary Table S1.

Although no statistically significant pre–post difference was observed, post-treatment plexus-depth values showed marked dispersion. This variability may reflect between-lesion heterogeneity, extreme observations, and/or measurement-related variability and therefore requires cautious interpretation of the group-level result. The absence of a statistically significant difference should not be interpreted as evidence of equivalent pre- and post-treatment plexus depth.

### 3.4. LC-OCT-Based Functional and Structural Vascular Parameters

**Figure 3:**
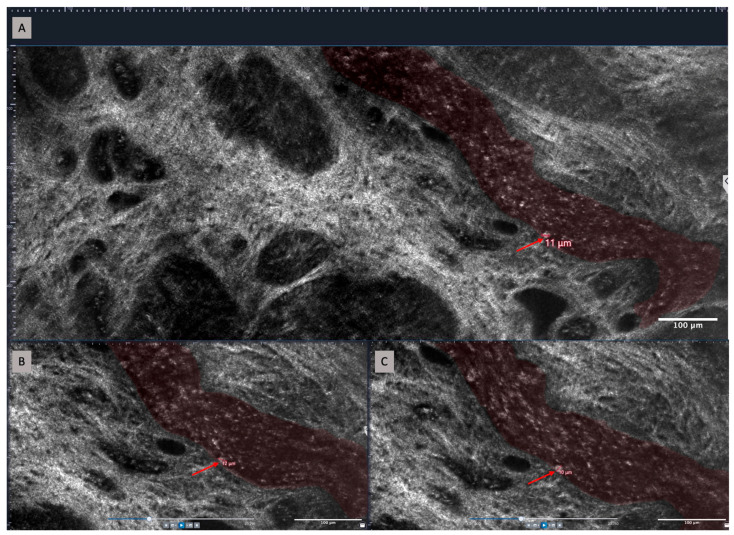
LC-OCT visualization of vessel-wall-associated intraluminal structures showing a rolling-like motion pattern in tumor-associated vessels in a basal cell carcinoma. (**A**) Overview of a dilated intratumoral vessel with a slowly moving cell-like structure adjacent to the vessel wall. (**B**,**C**) Sequential frames from the same LC-OCT sequence show movement of the structure along the vessel wall. LC-OCT morphology alone does not permit definitive cellular identification.

**Figure 4:**
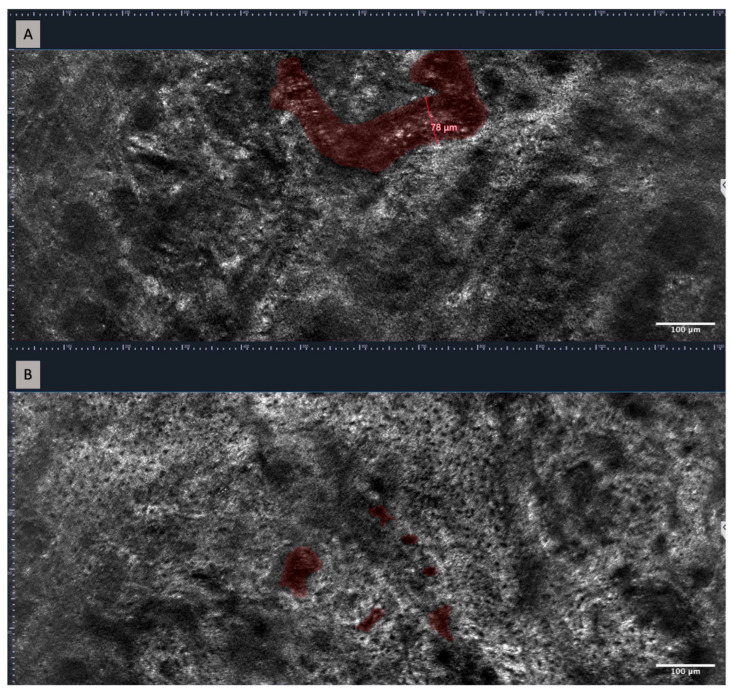
LC-OCT-based visualization of structural and functional vascular changes in a basal cell carcinoma following topical imiquimod therapy. (**A**) Pre-treatment images show irregularly configured, dilated vessels within tumor-associated tissue structures. (**B**) Sixteen weeks after completion of imiquimod therapy, narrowed vessels and a regular tissue architecture are observed, with no LC-OCT-visible typical tumor-associated structures, showing normalization of the assessed LC-OCT features, without implying histologically confirmed tumor clearance.

**Figure 5:**
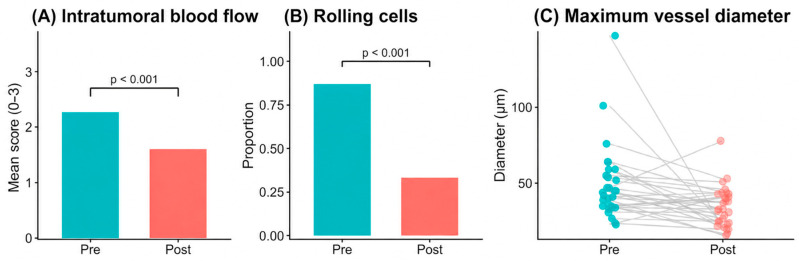
LC-OCT-based changes in vascular parameters in basal cell carcinomas before and after completion of topical imiquimod therapy. (**A**) Change in semiquantitative apparent intratumoral blood flow (LC-OCT video-based assessment) (score 0–3) between the pre-treatment examination (Pre) and follow-up after (Post) therapy. (**B**) Proportion of lesions showing vessel-wall-associated intraluminal structures with a rolling-like motion pattern on LC-OCT before (Pre) and after (Post) therapy. (**C**) Change in maximum vessel diameter (µm) between examination before (Pre) and after (Post) therapy. Each point represents one lesion with evaluable LC-OCT data at both time points (*n* = 30); gray lines connect paired measurements. Nominally significant lesion-level reductions were observed for the parameters shown (all *p* < 0.05). The displayed *p*-values refer to the primary lesion-level paired rank-based analyses. A cluster-adjusted paired change-score sensitivity analysis for LC-OCT maximum vessel diameter is reported in Supplementary Table S1. No cluster-adjusted mixed-effects result is reported for apparent intratumoral flow or for the binary rolling-like endpoint.

Exploratory video-based vascular features and structural vascular parameters were assessed before and after treatment using LC-OCT. Semiquantitatively assessed apparent intratumoral flow showed a nominally significant lesion-level time effect, with lower ordinal flow scores at follow-up (ATS = 13.285, df = 1, *p* < 0.001). Representative LC-OCT images of vessel-wall-associated intraluminal structures showing a rolling-like motion pattern are shown in Figure 3. The proportion of lesions showing vessel-wall-associated intraluminal structures with a rolling-like motion pattern decreased from 86.7% (26/30) before treatment to 33.3% (10/30) at follow-up (ATS = 13.357, df = 1, *p* < 0.001). The attempted patient-random-intercept logistic mixed-effects model for this binary endpoint had a singular/boundary fit; therefore, no regression-derived odds ratio or confidence interval is reported. Maximum vessel diameter also showed a nominally significant lesion-level time effect (ATS = 6.110, df = 1, *p* = 0.013). In the patient-cluster-adjusted paired change-score sensitivity analysis, the estimated mean change was −17.81 µm (95% CI: −34.40 to −1.23; *p* = 0.037; 30 paired lesions from 19 patients), and the model converged without a singular fit. This estimate was directionally consistent with the primary lesion-level analysis. Representative LC-OCT images illustrating structural and vascular changes before and after imiquimod therapy are shown in Figure 4. The lesion-level ATS analyses of apparent intratumoral flow and rolling-like status were not adjusted for clustering of multiple lesions within patients and may therefore underestimate uncertainty. All reported *p*-values are nominal and exploratory because no adjustment for multiple testing was applied to the main pre–post analyses. The paired pre-treatment and follow-up results for apparent intratumoral flow, rolling-like status, and maximum vessel diameter are summarized in Figure 5.

## 4. Discussion

Prior pilot work showed that LC-OCT may reveal residual BCC structures after imiquimod despite apparent clinical remission [3]. The present study extends this work by exploring treatment-associated vascular changes using complementary non-invasive modalities.

LC-OCT showed exploratory pre–post changes in apparent intratumoral flow and vascular morphology. The observed reduction in apparent flow is compatible with treatment-associated modulation of the tumor microenvironment. This observation is biologically plausible, as imiquimod, a TLR7 agonist, induces the release of antiangiogenic cytokines such as type I interferons and IL-12 and inhibits proangiogenic signaling pathways. This can lead to a functional remodeling of the tumor vasculature, including changes in vascular perfusion and the local microenvironment [25,26,27,28]. Unlike D-OCT, LC-OCT does not provide established quantitative perfusion metrics. Therefore, the LC-OCT-derived flow score and the assessment of vessel-wall-associated intraluminal structures showing a rolling-like motion pattern used in this study should be regarded as exploratory, semiquantitative video-based observations. Nevertheless, the high spatial resolution of LC-OCT enabled direct visualization of intravascular cellular movement and vessel-wall-associated structures, offering complementary insights into treatment-associated vascular remodeling.

The occurrence of vessel-wall-associated intraluminal structures showing a rolling-like motion pattern in LC-OCT decreased from 86.7% before treatment to 33.3% after treatment. The lesion-level rank-based analysis yielded a nominally significant time effect (ATS = 13.357, df = 1, *p* < 0.001). The leukocyte-adhesion literature supports the biological plausibility of slowly moving, wall-adjacent intraluminal cells in inflammatory microvascular processes [23]. However, these studies do not validate LC-OCT as a method for definitive cellular identification. Accordingly, the present finding should be interpreted as a treatment-associated change in an exploratory intravascular motion phenotype rather than as direct evidence of reduced leukocyte rolling. Alternative cellular or non-cellular intraluminal structures and imaging-related phenomena cannot be excluded. In the context of imiquimod therapy, it has been shown that BCC regression is associated with strong activation of the innate immune response, which makes the observed changes in the tumor microenvironment plausible [29].

In addition to the functional effects, a structural vascular change was also observed in LC-OCT. The descriptive lesion-level mean change in LC-OCT maximum vessel diameter was −14.4 µm. The corresponding paired rank-based analysis demonstrated a nominally significant time effect (ATS = 6.110, df = 1, *p* = 0.013). The additional patient-cluster-adjusted change-score sensitivity analysis yielded an estimated mean change of −17.81 µm (95% CI: −34.40 to −1.23; *p* = 0.037). This finding is consistent with the antiangiogenic effect of imiquimod described in the literature, which can lead to a modulation of tumor vascularization via the induction of antiangiogenic cytokines and the inhibition of proangiogenic signaling pathways [25].

The dermoscopic shift toward smaller vessel-diameter categories is consistent with earlier reports of regression of arborizing vessels during imiquimod therapy [30]. Persistent arborizing vessels have also been associated with residual disease after non-ablative treatment [31]. Thus, the observed change may reflect superficial vascular regression, although dermoscopy cannot assess functional perfusion or deeper vascular remodeling.

Furthermore, no nominally significant pre-post changes in the vascular parameters assessed by D-OCT, including vascular density, vessel diameter, and depth of the subepidermal vascular plexus, were observed before and after completion of imiquimod therapy (all *p* > 0.05). Comparable studies on the systematic analysis of D-OCT-based vascular changes before and after imiquimod therapy are currently limited. The available literature on OCT during imiquimod treatment is limited to conventional OCT examinations focusing on morphological tumor parameters. For example, Banzhaf et al. demonstrated that OCT can detect structural changes in BCC during imiquimod treatment and rule out clinically suspected residual disease, without, however, analyzing dynamic or vascular parameters [9]. The absence of statistically significant D-OCT changes cannot be attributed to a single cause. The standardized assessment at a depth of 300 µm may not have captured vascular remodeling occurring predominantly in more superficial or deeper tissue compartments. Although follow-up was deliberately performed after the visible acute inflammatory reaction had subsided, residual subclinical inflammatory hyperemia may have persisted and could have partially compensated for or obscured a treatment-associated reduction in tumor-related vascular signals. Protocol-specific measurement variability, imaging artifacts, limited statistical power, and biological heterogeneity among lesions may also have contributed. The absence of statistically significant differences should not be interpreted as evidence of unchanged vascularity, as the study was not designed as an equivalence analysis and some post-treatment measurements showed marked dispersion. Because depth-resolved measurements, standardized inflammation scores, serial intermediate assessments, and formal test–retest data were not available, the relative contribution of these possible explanations cannot be determined. These interpretations therefore remain speculative and should be investigated in larger longitudinal studies using repeated depth-resolved D-OCT measurements and standardized measures of inflammation and image quality.

Changes in LC-OCT maximum vessel diameter did not differ significantly between the assigned response groups in the global analysis (H = 3.870, df = 2, raw *p* = 0.144; BH-adjusted *p* = 0.753), and all exploratory pairwise Δ comparisons were also non-significant (BH-adjusted *p* ≥ 0.173). The numerical gradient is descriptive and may be influenced by incorporation bias, observer-related non-independence, and the small, unbalanced groups; it does not show that vessel-diameter reduction predicts or validly tracks response. Future studies should prespecify the candidate endpoint and acquisition time points, use an independent blinded response standard with histological confirmation where appropriate, and prospectively record adherence, inflammation, image quality, and relevant lesion characteristics. Sample-size calculations should account for observed variability, the expected low proportion of non-responders, within-patient clustering, missing imaging data, and multiplicity. Any threshold or model should be developed with internal validation and then tested in an independent, preferably multicenter cohort before clinical utility is claimed.

### 4.1. Integration of Imaging Modalities

Dermoscopy, D-OCT, and LC-OCT provided complementary information: dermoscopy captured superficial morphology, LC-OCT showed exploratory functional and structural changes, and the selected D-OCT parameters showed no nominally significant pre–post differences. These modality-specific findings support multimodal rather than isolated interpretation.

High-frequency ultrasound (HFUS) represents an additional, comparatively widely available non-invasive modality for the assessment of BCC. It is primarily used to estimate tumor thickness, lateral and deep extension, and possible involvement of underlying structures, thereby supporting treatment planning. On gray-scale HFUS, BCC is generally visualized as a hypoechoic dermal lesion, although its contour, echogenicity, and internal morphology may vary according to histological subtype. When combined with color or power Doppler, HFUS can additionally demonstrate intratumoral and peritumoral vascular signals. The extent and distribution of these signals are heterogeneous; solid BCCs have been reported more frequently to show hypervascular patterns, whereas infiltrative tumors may more often appear hypovascular. These vascular patterns are not sufficiently specific to serve as stand-alone diagnostic or response criteria but may provide complementary information on tumor morphology and vascularity [32]. A recent prospective pilot study using a portable 20 MHz HFUS device demonstrated agreement between HFUS-derived and histopathologic BCC depth measurements and proposed a preliminary depth-correction equation [33,34]. Because this equation was derived from a small cohort and has not yet been externally validated, it should be interpreted cautiously. HFUS was not included in the present study; future multimodal studies should directly compare HFUS- and Doppler-derived structural and vascular findings with OCT-based parameters.

### 4.2. Limitations

This study has several limitations that must be taken into account when interpreting the results. The small sample size and the single-center study design limit the validity of the analyses and the generalizability of the results. Because of the small sample size, sparse endpoint categories, and the inclusion of multiple lesions from some patients, patient-level clustering could be addressed only in selected endpoint-specific sensitivity analyses. Cluster-adjusted paired change-score models were fitted for D-OCT plexus depth, D-OCT vessel density, D-OCT vessel diameter, and LC-OCT maximum vessel diameter. The models for D-OCT vessel density, D-OCT vessel diameter, and LC-OCT maximum vessel diameter converged, whereas the D-OCT plexus-depth model yielded a boundary/singular fit with the patient-level random-intercept variance estimated near zero. For LC-OCT apparent intratumoral flow, no ordinal mixed-effects model was fitted because of the small sample size and the sparse or uneven distribution of the ordinal categories. The attempted logistic mixed-effects model for the rolling-like binary endpoint also yielded a singular/boundary fit. Results for endpoints without a stable cluster-adjusted model were therefore based on lesion-level analyses, which may underestimate uncertainty when multiple lesions originate from the same patient. All findings should consequently be interpreted as exploratory.

A further limitation is that LC-OCT-based apparent-flow assessment and the evaluation of vessel-wall-associated intraluminal structures are unvalidated, observer-dependent video-based measures. Primary evaluation was performed by a single investigator who was aware of the examination time point, while selected second-reader review was neither systematic nor blinded. Time-point awareness may therefore have influenced both video-segment selection and semiquantitative ratings and may have led to overestimation of the observed pre–post differences. In addition, LC-OCT morphology cannot provide histological, immunohistochemical, or immunophenotypic confirmation of the identity of the rolling-like intraluminal structures; other cells, debris, aggregates, flow-related phenomena, or imaging artifacts cannot be excluded. Future studies should use standardized acquisition and segment-selection protocols, randomly ordered time-point-masked sequences, independent evaluation by multiple readers, formal interobserver and intraobserver agreement analyses, and correlation with validated vascular imaging or histological and immunohistochemical reference measures.

Systematic histological correlation was not performed because the study aimed to evaluate a fully non-invasive treatment and monitoring strategy for BCC treated with imiquimod. Furthermore, it should be noted that inflammatory reactions under imiquimod can influence image quality, particularly in OCT-based procedures. This meant that a standardized and comparable evaluation of vascular parameters during the active treatment phase was only possible to a limited extent. For this reason, additional interim measurements during ongoing therapy were not performed.

Furthermore, topical imiquimod therapy was administered in a real-world clinical setting without formal monitoring or documentation of application. This approach was deliberately chosen to reflect a treatment scenario as close to real-world practice as possible. Differences in individual compliance can therefore not be ruled out and may have contributed to interindividual variability in the observed effects. The lack of adherence monitoring represents a potential source of bias, as treatment intensity may have differed between patients. Local inflammatory reactions, such as erythema or crust formation, were recorded during follow-up and may serve as indirect indicators of treatment activity. However, these findings were not present in all cases and were not assessed using a standardized scoring system, limiting their value as reliable markers of treatment adherence. Undocumented differences in treatment intensity may have influenced both the magnitude of vascular changes and the assigned clinical/imaging response category. Because treatment exposure may therefore have affected both variables, adherence represents a potential unmeasured confounder in the response-stratified analysis. In particular, the available data do not allow true pharmacological non-response to be distinguished from inadequate or interrupted treatment exposure. This may have increased within-group heterogeneity and may have weakened, strengthened, or otherwise distorted the descriptive associations between vascular changes and clinical/imaging response. Accordingly, the response-oriented analysis should not be interpreted as a direct assessment of the pharmacological efficacy of imiquimod or as evidence of intrinsic treatment resistance.

Another limitation is the lack of a formal interobserver agreement analysis. Because the evaluators were not blinded to the pre- or post-treatment time point, confirmation bias cannot be excluded, particularly for semiquantitative LC-OCT parameters such as the apparent-flow score and the assessment of vessel-wall-associated cellular structures. Awareness of the examination time point may have influenced both the selection of representative video segments and the semiquantitative assessment of apparent flow and vessel-wall-associated cellular structures. Consequently, the magnitude of the observed pre–post differences may have been overestimated. Image evaluation was primarily performed by a single investigator, which may introduce observer-dependent bias. Although selected datasets were reviewed by a second experienced investigator for quality assurance, the reproducibility of the semiquantitative LC-OCT-based parameters was not formally assessed. Future studies should use randomly ordered, time-point-masked video sequences evaluated independently by at least two readers, with formal assessment of interobserver agreement.

The response-stratified analysis was exploratory and was not powered for comparisons between the small and unbalanced response groups. Treatment response was not systematically confirmed histologically. Consequently, subclinical residual tumor may have been present in lesions classified as showing complete clinical/imaging response. Normalization of vascular imaging parameters therefore cannot be equated with histologically confirmed tumor clearance and may reflect remodeling of the local microenvironment despite persistent tumor cells. Such misclassification could have strengthened, weakened, or otherwise distorted the observed descriptive relationship between vascular changes and the assigned response category. Because response classification partly incorporated imaging findings and was not based on an independent blinded reference standard, incorporation bias and observer-related non-independence may additionally have influenced the results. The response-stratified analysis should therefore be interpreted as an exploratory association with the assigned clinical/imaging response category rather than as evidence of diagnostic performance for residual tumor. Future validation studies should use an independent, blinded reference standard, including histological confirmation where clinically and ethically appropriate. Future studies with combined histological and imaging follow-up could help further validate the significance of the observed vascular changes.

Furthermore, the post-treatment examination was conducted at a single time point between 12 and 16 weeks after completion of therapy. Since only two time points were recorded, this does not constitute a true longitudinal follow-up. Conclusions regarding the temporal dynamics of vascular changes during ongoing therapy are therefore not possible.

Different histological subtypes of basal cell carcinoma (superficial, nodular, and infiltrative BCC) were included. These subtypes differ in part with regard to their biological properties and vascular architecture, so that a certain degree of biological heterogeneity among the examined lesions cannot be ruled out. Overall, the exploratory nature of the study should be emphasized, meaning that the results should primarily be viewed as hypothesis-generating and should be further validated in larger, prospective studies with standardized protocols. In particular, the small and unbalanced distribution of the response groups, with only a few lesions showing no response, limits the interpretability of the response-oriented supplementary analyses.

## 5. Conclusions

In this exploratory prospective observational study, LC-OCT detected nominally significant functional and structural vascular changes after topical imiquimod therapy, whereas D-OCT parameters remained largely unchanged and dermoscopy showed reduced superficial vessel caliber. These findings suggest that LC-OCT may provide complementary information for non-invasive assessment of treatment-associated vascular remodeling in BCC. Owing to the small sample size, heterogeneous histological subtypes, lack of systematic post-treatment histology, and exploratory statistical approach, the results should be interpreted as hypothesis-generating and require validation in larger prospective studies. The findings support further prospective evaluation of LC-OCT-derived vascular features as exploratory complementary parameters during non-invasive follow-up; they do not yet establish a validated marker of treatment response.

## Figures and Tables

**Table 1:** Patient and lesion characteristics.

Characteristic	Value
Patients, *n*	20
Female/male, *n*	9/11
Age, mean ± SD (years)	72.3 ± 8.6
Age, median (range, years)	73 (51–93)
Lesions, *n*	31
**BCC subtype ^1^, *n*/*N* (%)**	
Superficial BCC	23/30 (76.7%)
Nodular BCC	6/30 (20.0%)
Infiltrative BCC	1/30 (3.3%)
Missing subtype	1/31 (3.2%)
Tumor thickness (mm), mean ± SD	0.73 ± 0.23
**Location, *n*/*N* (%)**	
Head/neck	2/31 (6.5%)
Face	8/31 (25.8%)
Trunk	8/31 (25.8%)
Extremities	13/31 (41.9%)
**Risk zone ^2^, *n*/*N* (%)**	
Low	20/31 (64.5%)
Intermediate	5/31 (16.1%)
High	6/31 (19.4%)
Pigmentation, *n*/*N* (%)	3/31 (9.7%)
Ulceration, *n* (%)	13/31 (41.9%)
**Diagnostic confirmation, *n*/*N* (%)**	
Diagnostic biopsy before treatment	5/31 (16.1%)

^1^ BCC subtype was available for 30 lesions; one value was missing. Subtype was determined histologically where available and otherwise by non-invasive imaging. ^2^ Risk zones were defined according to anatomical location: low-risk (trunk and extremities), intermediate-risk (cheeks, forehead, scalp, and neck), and high-risk (central face [including nose, eyelids, periorbital area, lips], ears, temples, and genital area).

**Table 2:** Selectionlesion-level changes in LC-OCT endpoints by assigned clinical/imaging response group ^1^.

Endpoint	Group	*n*	Δ Mean (SD)	Δ Median [IQR]	Δ Range
Maximum vessel diameter ^1^	No response	3	3.33 (6.66)	5.00 [0.50; 7.00]	−4.00 to 9.00
	Partial response	9	−9.44 (12.49)	−4.00 [−15.00; −1.00]	−36.00 to 3.00
	Complete response	18	−19.83 (35.36)	−13.50 [−23.00; −3.50]	−126.00 to 31.00
Rolling-like status ^2^	No response	3	−0.33 (0.58)	0.00 [−0.50; 0.00]	−1.00 to 0.00
	Partial response	9	−0.44 (0.53)	0.00 [−1.00; 0.00]	−1.00 to 0.00
	Complete response	18	−0.61 (0.50)	−1.00 [−1.00; 0.00]	−1.00 to 0.00

^1^ This response-stratified analysis was exploratory and descriptive. Response classification was not based on an independent, blinded reference standard and partly incorporated imaging findings, resulting in potential incorporation bias. For the change in LC-OCT maximum vessel diameter, the global Kruskal–Wallis comparison was not statistically significant (H = 3.870, df = 2, raw *p* = 0.144; BH-adjusted *p* = 0.753). All exploratory pairwise Δ comparisons were also non-significant (BH-adjusted *p* ≥ 0.173). ^2^ The presence of LC-OCT vessel-wall-associated intraluminal structures showing a rolling-like motion pattern was recorded as a binary variable (0 = absent, 1 = present). The reported change represents the difference between post-treatment follow-up and pre-treatment measurements (Δ = post-treatment follow-up − pre-treatment), with negative values indicating a decrease over time.

**Table 3:** Metric vascular D-OCT parameters before and after imiquimod therapy.

Parameter	Pre, Median (Q1–Q3)	Post, Median (Q1–Q3)	Paired *n*	*p*
Plexus depth (µm)	189.5 (124.2–274.2)	228.5 (148.2–393.2)	30	0.555
Vessel diameter (µm)	65.5 (49.0–85.5)	69.5 (42.2–101.8)	26	0.758
Vessel density (%)	7.0 (4.0–12.0)	6.8 (3.0–14.8)	30	0.902

Table legend: Metric vascular parameters measured using dynamic optical coherence tomography (D-OCT) are presented as median and interquartile range (IQR) because of skewed distributions. The paired n indicates the number of lesions with evaluable measurements at both time points. *p*-values in this table refer to the primary lesion-level rank-based paired analyses and are not adjusted for clustering of multiple lesions within patients. Additional cluster-adjusted paired change-score sensitivity analyses for D-OCT plexus depth, D-OCT vessel density, and D-OCT vessel diameter are provided in Supplementary Table S1. These analyses used complete paired lesion-level cases and linear mixed-effects models of Post-minus-Pre change scores with a random intercept for patient. Model estimates, 95% confidence intervals, *p*-values, and boundary or singular fits are reported in the Supplementary Table S1.

**Table 4:** Semiquantitative D-OCT parameters before and after imiquimod therapy.

Parameter (Ordinal)	Pre-Distribution (*N* = 30)	Post-Distribution (*N* = 30)	*p*
Vessel density (0–2)	0: 20%/1: 53.3%/2: 26.7%	0: 33.3%/1: 53.3%/2: 13.3%	0.234
Vessel diameter (0–2)	0: 20%/1: 56.7%/2: 23.3%	0: 30%/1: 50%/2: 20%	0.576

Table legend: Semiquantitative D-OCT parameters for assessing vascular density and vessel diameter before and after treatment. The table shows the percentage distributions of the ordinal categories. The *p*-values refer to the before-and-after comparison and were obtained from lesion-level analyses without adjustment for clustering of multiple lesions within patients and may therefore be anti-conservative. These results are interpreted as exploratory.

**Table 5:** Qualitative Vascular Morphology in D-OCT Before and After Imiquimod Therapy.

Characteristic	Pre, *n* (%) (*N* = 30)	Post, *n* (%) (*N* = 30)	*p*
Inflammation	23 (76.7)	25 (83.3)	0.821
Coils	5 (16.7)	4 (13.3)	0.781
Lines	25 (83.3)	25 (83.3)	0.970
Curves	19 (63.3)	17 (56.7)	0.944
Serpiginous	20 (66.7)	17 (56.7)	0.263
Branching	17 (56.7)	16 (53.3)	0.618
Columns	19 (63.3)	11 (36.7)	0.057

Table legend: Qualitative vascular morphological features on D-OCT before and after treatment. The table shows the absolute frequencies and percentages of the lesions examined. The *p*-values refer to the before-and-after comparison and were obtained from lesion-level analyses without adjustment for clustering of multiple lesions within patients and may therefore be anti-conservative. These results are interpreted as exploratory.

## Data Availability

The data presented in this study are available from the corresponding author upon reasonable request. Due to privacy and ethical restrictions, patient-level imaging data are not publicly available.

## Supplementary Materials

The following supporting information can be downloaded at: https://www.mdpi.com/article/10.3390/cancers18132153/s1, Supplementary Table S1: Patient-cluster-adjusted paired change-score sensitivity analyses for continuous imaging endpoints. For each lesion, change was defined as Δ = follow-up minus pre-treatment. Models were fitted using lmerTest::lmer(delta ~ 1 + (1 | patient_id), REML = TRUE), with a random intercept for patient. The fixed intercept estimates the mean lesion-level change, with negative estimates indicating a reduction at follow-up. Ninety-five percent confidence intervals were calculated as Estimate ± $t_{0.975,df}$ × SE using the endpoint-specific denominator degrees of freedom provided by lmerTest. *p*-values were obtained from summary(fit)$coefficients and correspond to the lmerTest/Satterthwaite output. Singularity was assessed using lme4::isSingular(fit, tol = 1 × 10^−5^). These models were performed as sensitivity analyses and do not replace the primary lesion-level paired rank-based analyses. Singular or boundary fits are reported transparently and interpreted cautiously. Supplementary Table S2: Exploratory lesion-level response-group comparisons for pre-treatment, follow-up, and change values. Table S2A. Global Kruskal-Wallis-Tests. Table S2B. Exploratory pairwise comparisons of Δ values.

## Abbreviations

The following abbreviations are used in this manuscript:

BCC	Basal cell carcinoma
sBCC	Superficial basal cell carcinoma
nBCC	Nodular basal cell carcinoma
iBCC	Infiltrative basal cell carcinoma
D-OCT	Dynamic optical coherence tomography
LC-OCT	Line-field confocal optical coherence tomography
OCT	Optical coherence tomography
TLR7	Toll-like receptor 7
5-FU	5-Fluorouracil
EMA	European Medicines Agency
ATS	ANOVA-type statistic
SD	Standard deviation
µm	Micrometer
