# Supplementary material for: Exploratory Multimodal Analysis of Vascular Changes in Basal Cell Carcinoma Before and After Topical Imiquimod Therapy Using Dermoscopy and Non-Invasive Imaging
Source: Cancers (Basel). 2026 Jul 4;18(13):2153. doi: 10.3390/cancers18132153 (PMC13360017; doi:10.3390/cancers18132153)
Supplement: Supplementary file 1 [file cancers-18-02153-s001.zip › cancers-4375509-supplementary.pdf]

cancers

MDPI

Supplementary Materials:

Supplementary Table S1. Patient-cluster-adjusted paired change-score sensitivity analyses for continuous imaging endpoints. For each lesion, change was defined as Δ = follow-up minus pre-treatment. Models were fitted using `lmerTest::lmer(delta ~ 1 + (1 | patient_id), REML = TRUE)`, with a random intercept for patient. The fixed intercept estimates the mean lesion-level change, with negative estimates indicating a reduction at follow-up. Ninety-five percent confidence intervals were calculated as Estimate ± $t_{0.975,df}$ × SE using the endpoint-specific denominator degrees of freedom provided by lmerTest. P-values were obtained from `summary(fit)$coefficients` and correspond to the lmerTest/Satterthwaite output. Singularity was assessed using `lme4::isSingular(fit, tol = 1e-5)`. These models were performed as sensitivity analyses and do not replace the primary lesion-level paired rank-based analyses. Singular or boundary fits are reported transparently and interpreted cautiously.

Table S1.

**Endpoint**	**Paired lesions, n**	**Patients, n**	**Estimated mean change, Post minus Pre (95% CI)**	**p-value**	**Model status**
D-OCT plexus depth (µm)[1]	30	20	159.83 (−115.40 to 435.07)	0.245	Boundary/singular fit; patient-level random-intercept variance estimated near zero
D-OCT vessel density (%)[2]	30	20	1.48 (−3.19 to 6.16)	0.515	Model converged
D-OCT vessel diameter (µm)	26	19	7.94 (−12.53 to 28.42)	0.428	Model converged
LC-OCT maximum vessel diameter (µm)	30	19	−17.81 (−34.40 to −1.23)	0.037	Model converged

[1] The D-OCT plexus-depth result should be interpreted cautiously because the patient-level variance component was estimated near zero.

[2] For D-OCT vessel density, the estimate represents the absolute change in percentage points.

Supplementary Table S2. Exploratory lesion-level response-group comparisons for pre-treatment, follow-up, and change values.

Table S2A. Global Kruskal-Wallis-Tests

**Endpoint**	**Pre raw p**	**Pre BH p**	**Follow-up raw p**	**Follow-up BH p**	**Δ raw p**	**Δ BH p**
Dermoscopy diameter	0.795	0.876	0.291	0.723	0.393	0.753
LC-OCT rolling-like status	0.573	0.859	0.429	0.723	0.558	0.753
Blood-cell diameter, centre	0.372	0.764	0.502	0.723	0.378	0.753
Blood-cell diameter, marginal	0.382	0.764	0.459	0.723	0.628	0.753
Apparent intratumoral flow	0.876	0.876	0.958	0.958	0.964	0.964
Maximum vessel diameter	0.152	0.764	0.603	0.723	0.144	0.753

Table S2B. Exploratory pairwise comparisons of Δ values

Endpoint	No vs partial response	No vs complete response	Partial vs complete response
Dermoscopy diameter	0.423	0.423	0.423
LC-OCT rolling-like status	0.829	0.656	0.656
LC-OCT blood-cell diameter, centre	0.497	0.686	0.497
LC-OCT blood-cell diameter, marginal	0.850	0.813	0.813
LC-OCT apparent intratumoral flow	1.000	1.000	1.000
LC-OCT maximum vessel diameter	0.173	0.173	0.381

Global response-group comparisons were performed separately for pre-treatment, follow-up, and lesion-level change values using `stats::kruskal.test`. Within each analysis set, global p-values were adjusted across the six endpoints using the Benjamini–Hochberg method. Pairwise comparisons of lesion-level Δ values were performed using `pairwise.wilcox.test(..., p.adjust.method = "BH", exact = FALSE)`. Reported values are Benjamini–Hochberg-adjusted p-values across the three pairwise group comparisons within each endpoint. Because none of the global Kruskal–Wallis tests for Δ values was statistically significant, the pairwise analyses are presented for completeness and should be interpreted as exploratory.
